# Mental health issues and their association with sleep quality among women of reproductive age in Noakhali, Bangladesh

**DOI:** 10.1371/journal.pgph.0005742

**Published:** 2025-12-30

**Authors:** Towhid Hasan, Nahian Rahman, Abu Zafar Md Saleh, Md Didar Hossain, Marjia Sultana

**Affiliations:** Department of Food Technology and Nutrition Science, Noakhali Science and Technology University, Noakhali, Bangladesh; University of Tunis El Manar Faculty of Medicine of Tunis: Universite de Tunis El Manar Faculte de Medecine de Tunis, TUNISIA

## Abstract

Mental health issues like anxiety and depression are prevalent worldwide, particularly affecting women of reproductive age (WRA). These conditions may impact sleep quality, which is crucial for overall well-being. However, no prior study in Bangladesh has examined the relationship between anxiety, depression, and sleep quality specifically among this demographic. Hence, this study aimed to assess anxiety and depressive symptoms and their association with sleep quality among WRA in Noakhali, Bangladesh. A cross-sectional study was conducted from May to June 2024 among 417 women aged 15–49 years in Noakhali, Bangladesh. Data were collected through a structured questionnaire assessing socio-demographic characteristics, anxiety (Generalized Anxiety Disorder [GAD-7]), depression (Patient Health Questionnaire [PHQ-9]), and sleep quality (Pittsburgh Sleep Quality Index [PSQI]). The prevalence of anxiety symptoms, depressive symptoms, and poor sleep quality among participants was 44.6%, 47.0%, and 36.9%, respectively. Women with anxiety and depressive symptoms had significantly higher PSQI scores than those without symptoms (p < 0.05). Multiple linear regression analysis revealed that higher anxiety (*β* = 0.243, p < 0.001) and depressive (*β* = 0.285, p < 0.001) symptoms scores were associated with poorer sleep quality. In conclusion, a high burden of anxiety, depression, and poor sleep quality is evident among WRA in Noakhali. This study provides the first evidence from Bangladesh highlighting the strong link between mental health and sleep quality among WRA, underscoring the need for integrated mental and sleep health interventions in rural and underserved communities.

## Introduction

Worldwide, approximately 792 million (10.7%) people are suffering from mental health issues [1]. Among these conditions, anxiety and depression are the most prevalent and ranked among the 25 leading causes of global burden [2,3]. It is expected that globally, anxiety and depressive disorders will be the 19^th^ and 7^th^ leading cause of disease burden by 2050 [3]. Anxiety is marked by excessive tension and persistent fear, often causing overwhelming fear, substantial distress, functional impairment, and development of other mental health conditions like depression when severe or prolonged. [4]. Depression is characterized by long-term feelings of sadness, hopelessness, and loss of pleasure in activities once enjoyed. Both of these issues may lead to significant morbidity, disability, decreased productivity, and financial strain [5].

Women are experiencing the symptoms of anxiety and depression almost twice as likely as men with their global prevalence estimated at around 4.6% and 5.1%, respectively [6]. In South Asian countries, including Bangladesh, Nepal, and India, mental health issues among women remain a significant concern, with prevalence rates varying across regions but consistently indicating substantial burden [1,3,5]. However, in low and middle-income countries like Bangladesh, these conditions are often underreported and undertreated due to stigma, lack of awareness, and limited access to healthcare services, particularly in rural and underserved areas [7].

Women of reproductive age (WRA), typically between 15 and 49 years, represent a crucial demographic group due to their role in fertility, maternal health, and family well-being [8]. This life stage is marked by significant biological, social, and economic responsibilities, making their health and well-being essential for both individual and societal development [9]. Women in this age group experience significant physiological, psychological, and social challenges [10].

Physiological changes encompass critical reproductive events such as puberty, menstruation, pregnancy, childbirth, and menopause [6]. Besides they often face multiple stressors, including economic burdens, family responsibilities, and societal pressures as social challenges [6,9]. These challenges are often amplified in rural settings where women may face additional barriers such as restricted mobility, limited economic opportunities, reduced access to education and healthcare, and stronger adherence to traditional gender roles that can restrict help-seeking behaviors [7]. All of the above challenges influence their overall well-being making them more susceptible to mental health disorders such as anxiety, depression, and so on [10]. Consequently, anxiety and depression faced by women belonging to the reproductive age group may further impair their daily functioning, increase the risk of sleep disturbance, enhance tiredness and fatigue, minimize productivity and appetite, and compromise the overall quality of life [1,4].

Sleep disturbances, a common manifestation of anxiety and depression [11], are often responsible for the disruption of hormonal balance, menstrual irregularities, reduction of fertility, and complications that arise during pregnancy including gestational diabetes, preterm birth, and preeclampsia among WRA [12–14]. Additionally, inadequate sleep is associated with increased levels of stress hormones like cortisol, which can worsen their emotional well-being [15]. Cognitive impairment, including reduced attention, memory issues, and difficulty in decision-making, is also commonly reported due to poor sleep quality which affects their daily productivity [16]. Furthermore, long-term sleep deprivation weakens immune function, making them more susceptible to infections and chronic conditions such as obesity, diabetes, and cardiovascular diseases. Consequently, poor sleep patterns can lead to fatigue, mood swings, and decreased resilience to stress, increasing the risk of postpartum depression and other mental health disorders [15]. Thus, addressing sleep disturbances in this population is of urgent need to maintain their reproductive health, emotional stability, and overall well-being.

Given the importance of mental health and sleep in ensuring the well-being and productivity of WRA, it is crucial to investigate their interrelationship in this demographic group. There are few existing studies on mental health and sleep quality that largely focus on the general population such as adults [17] or high-risk populations such as patients [18,19], elderly [20], students [21], pregnant women [22], etc. with insufficient attention given to WRA who experiencing unique physiological and psychosocial challenges. However, no study has examined the association of sleep quality with anxiety and depression symptoms among this population group, particularly in Bangladesh. Thus, this study aimed to assess anxiety and depression and their impact on sleep quality among WRA in Noakhali, Bangladesh. Understanding these associations is also essential for designing effective interventions that will help to promote their mental well-being and sleep health.

## Methods

### Ethics statement

The study protocol was reviewed and approved by the Ethical Committee of Noakhali Science and Technology University (Ref no.: NSTU/SCI/EC/2024/273). Both oral and written informed consent was obtained from the participants and parents or guardians of the minors (aged below 18 years) before data collection.

### Study design, settings, and population

A cross-sectional study was carried out among women aged 15–49 years living in five upazilas (subdistricts) of Noakhali (Maijdee, Senbag, Subarnachar, Kabirhat, Sonaimuri), Bangladesh from 30/05/2024–15/06/2024. Noakhali district was chosen as the study site because it represents a typical rural and coastal setting in Bangladesh, characterized by moderate to low socio-economic status, high rates of female labor participation in informal sectors, and limited access to healthcare services, including mental health care. A household listing was obtained from local administrative offices (Union Parishad) to create a comprehensive registry of eligible households containing women aged 15–49 years. From this registry, households were randomly selected using a random number generator. The sample size was calculated using Cochran’s formula [23,24] of n = z^2^p(1–p)/d^2^, with a 95% confidence level (z = 1.96), a margin of error of 5% (d = 0.05), and an assumed population proportion of 50% (p = 0.5). After considering a 5% non-response rate, the total sample size was 405. However, information was collected from 417 subjects for better confidence. Women aged less than 15 or older than 49 years, and those unable to participate due to acute illness were excluded from this study.

### Study instrument

A structured questionnaire comprising both open- and close-ended questions was first designed based on previous studies [22,25,26]. The draft questionnaire was reviewed by two public health experts. A pilot study was also conducted with 20 women from the same study population (5% of the total sample size). Following expert review and pilot study, necessary modifications were incorporated and the final questionnaire was prepared.

#### Socio-demographic and anthropometric assessment.

Socio-demographic data included age, residence, marital status, level of education, occupation, monthly household income and family size. The anthropometric measurements included weight and height. Weight was measured using a digital weighing scale (SECA 813, Hamburg, Germany) to the nearest 0.1 kg, and height was measured using a portable stadiometer (SECA 213, Hamburg, Germany) to the nearest 0.1 cm. Body mass index (BMI) was calculated by dividing the weight in kilograms (kg) by the height in meters squared (m^2^). Participants were categorized as undernutrition, normal nutritional status, overweight, pre-obese, and obese when the BMI was < 18.5, 18.5–22.9, 23–24.9, 25-29.9, and ≥ 30 kg/m^2^ [27].

#### Assessment of dietary diversity.

The minimum dietary diversity for women (MDD-W) was used to assess the overall dietary diversity of WRA. The MDD-W indicator was based on a set of 10-food groups: 1) starchy staples group (grains, white roots and tubers, and plantains), 2) pulses (beans, peas, and lentils), 3) nuts and seeds, 4) dairy, 5) animal foods (meat, poultry, and fish), 6) eggs, 7) vitamin A rich dark green leafy vegetables, 8) other vitamin A rich fruits and vegetables, 9) other vegetables, and 10) other fruits. Women were asked to report all foods they had consumed from the listed food groups during the previous day. The answers were recorded as either ‘yes’ or ‘no’. A ‘yes’ was assigned a score of ‘1’, and a ‘no’ was assigned a score of ‘0’. These scores were then summed to calculate an overall score. Women with a score below 5 were categorized as having low dietary diversity, while those with scores of 5 or higher were considered to have high dietary diversity [28].

#### Assessment of physical activity.

Physical activity was measured using the International Physical Activity Questionnaire - Short Form (IPAQ-SF), which evaluates activity over the previous week [29]. The 7-item questionnaire captures four levels of physical activity intensity:

- Vigorous activities (like heavy lifting, aerobics, fast cycling)
- Moderate activities (like light load carrying, regular cycling, doubles tennis)
- Walking
- Sitting

Physical activity was quantified as energy expenditure in metabolic equivalent tasks (MET)-minutes per week. Total METs were calculated using this formula:

(Vigorous activity minutes per day × days per week × 8.0) + (Moderate activity minutes per day × days per week × 4.0) + (Walking minutes per day × days per week × 3.3)

Participants were considered to have sufficient physical activity if they achieved at least 600 MET-minutes per week [29].

#### Assessment of anxiety.

Generalized Anxiety Disorder (GAD) scale, a self-administered 7-item questionnaire (GAD-7) was used to determine the anxiety disorder among the participants. The questionnaire has previously been validated and used in many studies in Bangladesh among different population groups [30,31]. In this measure, the participants were asked to rank their responses on how often they have experienced symptoms like feeling nervousness, anxiousness, etc., over the last two weeks using a 4 - points Likert scale (0 = not at all, 1 = several days, 2 = more than half of days and 3 = nearly every day). Summing up the scores of all the items, a total score ranging from 0 to 21 was observed, and the participants were categorized into two groups: without anxiety symptoms (GAD-7 score < 5) and with anxiety symptoms (GAD-7 score ≥ 5). Confirmatory factor analysis (CFA) indicated good structural validity, with good model fits in this sub-scale: χ^2^/*df* = 1.386, comparative fit index (CFI) = 0.992, Tucker-Lewis index (TLI) = 0.984, root mean square error of approximation (RMSEA) = 0.030, standardized root mean squared residual (SRMR) = 0.026. This scale was also reliable, with a Cronbach’s α coefficient of 0.705.

#### Assessment of depression.

A previously validated 9-item Patient Health Questionnaire (PHQ-9) was used to assess the occurrence and severity of depressive symptoms among participants over the past two weeks [31–33]. Each item was evaluated using a 4-point Likert scale (0 = not at all, 1 = several days, 2 = more than half of days, and 3 = nearly every day) with a total score of 0–27. The participants were categorized into two groups based on their PHQ-9 scores: having no depressive symptoms (score < 5) and having depressive symptoms (score ≥ 5). CFA presented good model fits in this scale: χ^2^/*df* = 1.689, CFI = 0.973, TLI = 0.958, RMSEA = 0.041, SRMR = 0.037. This scale had good internal consistency, with a Cronbach’s α coefficient of 0.712.

#### Sleep quality assessment.

Pittsburgh Sleep Quality Index (PSQI), a previously validated and used scale in Bangladesh was utilized to assess sleep quality among the participants [22,34,35]. This 19-item questionnaire measured subjective sleep quality, sleep latency, sleep duration, habitual sleep efficiency, sleep disturbances, use of sleeping medication, and daytime dysfunction. Each subscale weighted equal possible score of 0–3 and the global PSQI score ranged from 0 to 21. A global PSQI score of ≥ 5 indicated poor sleep quality. CFA indicated structural good validity, with model fits in this scale: χ^2^/*df* = 1.532, CFI = 0.988, TLI = 0.977, RMSEA = 0.036, SRMR = 0.029. The Cronbach’s α coefficient was 0.703, suggesting this scale was a reliable measurement tool.

### Data collection

The data collectors were graduate students from the Department of Food Technology and Nutrition Science, Noakhali Science and Technology University. They were trained by the principal investigator of this study in terms of data collection techniques, inclusion/exclusion criteria, etc. The data collectors described the objectives, benefits and potential risks of the study to each of the participants and ensured them that the privacy of their information would be maintained. Data were collected through a face-to-face interview that took around 15 min.

### Statistical analysis

This study assessed the validity and reliability of the scales using various statistical techniques. The study applied the following criteria to determine a scale’s goodness of fit: the comparative fit index (CFI) and Tucker-Lewis index (TLI) of ≥0.95, the root mean square error of approximation (RMSEA) of ≤0.06, and the standardized root mean square residual (SRMR) of <0.08 [36]. The scale’s fitness was also evaluated through the relative chi-square method, where a chi-square to degrees of freedom ratio (χ^2^/*df*) ≤2.00 indicated a good fit [37]. Additionally, Cronbach’s α coefficients were used to assess the internal reliability of each scale.

The socio-demographic, sleep quality, anxiety and depressive symptoms scores were presented by frequency, percentages, mean, and standard deviation (SD) where necessary. Spearman’s rank correlation was conducted to ascertain the relationship between overall sleep quality and each component score, while the association between the sleep quality scores with anxiety, and depressive symptoms was analyzed using the *t*-test. Additionally, a multiple linear regression analysis was performed in order to control the relationship between sleep quality and anxiety, and depressive symptoms. Scale and model fit analyses were conducted in SPSS AMOS (version 24.0) for Windows (IBM Co., Armonk, NY, USA), whereas all other analyses were done using SPSS (version 27.0) for Windows (IBM Co., Armonk, NY, USA). A P < 0.05 (two-tailed) was set as statistical significance.

## Results

Table 1 shows the socio-demographic and related characteristics of the participants. Around half of the participants were aged 20–29 years. Most of the participants resided in rural areas, were married, and primarily engaged as housewives. More than one-third of the participants had no formal schooling. Majority of the participants had monthly household income of >15000 Bangladeshi taka (BDT) and belonged to a family having more than 4 members. Around half of the participants were in normal nutritional status having a BMI 18.5–22.9 kg/m^2^. More than two-thirds of the women met the minimum dietary diversity (MDD-W ≥ 5) and all participants reported engaging in sufficient physical activity.

**Table 1:** Socio-demographic and related characteristics of the participants.

Variables	n (%)
*Age (years)*	
15-19	1 (0.2)
20-29	212 (50.9)
30-39	123 (29.5)
≥ 40	81 (19.4)
*Residence*	
Rural	384 (92.1)
Urban	33 (7.9)
*Marital status*	
Unmarried	7 (1.7)
Married	377 (90.4)
Divorced	10 (2.4)
Widowed	23 (5.5)
*Level of education*	
No formal schooling	164 (39.3)
Primary	144 (34.5)
Secondary	85 (20.4)
Tertiary	24 (5.8)
*Occupation*	
Housewife	386 (92.6)
Business	5 (1.2)
Private employee	21 (5.0)
Government employee	5 (1.2)
*Monthly household income (BDT)*	
≤ 15000	168 (40.3)
>15000	249 (59.7)
*Family size (persons)*	
≤ 4	102 (24.5)
>4	315 (75.5)
*BMI (kg/m*^*2*^)	
< 18.5	29 (7.0)
18.5–22.9	178 (42.7)
23.0–24.9	110 (26.4)
25.0–29.9	94 (22.5)
≥ 30	6 (1.4)
*MDD-W*	
< 5	121 (29.0)
≥ 5	296 (71.0)
*Physical activity*	
Sufficient	417 (100.0)

BDT: Bangladeshi taka, BMI: Body mass index, MDD-W: Minimum dietary diversity for women.

The assessment of anxiety symptoms among the participants are shown in Table 2. The majority of the participants (77.9%) reported feelings of nervousness, anxiety, or unease for several days. Almost half of the participants had difficulty in stopping or controlling worrying (54.4%) and relaxing (50.6%). Similarly, 52.8% experienced restlessness to the extent that sitting still was challenging, while 51.8% frequently became easily annoyed or irritable. Furthermore, 53.4% worried too much about different things and 46.8% expressed fear that something terrible might happen for several days to more than half the days. About 44.6% of the women reported experiencing anxiety symptoms.

**Table 2:** Assessment of anxiety symptoms among participants.

Item	Not at all (%)	Several days (%)	More than half the days (%)	Nearly everyday (%)
1. Feeling nervous, anxious, or on edge	60 (14.4)	325 (77.9)	32 (7.7)	–
2. Not being able to stop or control worrying	144 (34.6)	227 (54.4)	45 (10.8)	1 (0.2)
3. Worrying too much about different things	193 (46.3)	159 (38.1)	63 (15.1)	2 (0.5)
4. Trouble relaxing	181 (43.4)	211 (50.6)	24 (5.8)	1 (0.2)
5. Being so restless that it is hard to sit still	159 (38.1)	220 (52.8)	35 (8.4)	3 (0.7)
6. Becoming easily annoyed or irritable	142 (34.1)	216 (51.8)	58 (13.9)	1 (0.2)
7. Feeling afraid, as if something awful might happen	219 (52.5)	172 (41.3)	23 (5.5)	3 (0.7)
Total GAD-7 score (Mean±SD)	5.09 ± 2.67			

GAD-7: Generalized Anxiety Disorder.

Table 3 summarizes the prevalence of depressive symptoms among the participants. Majority of the participants reported experiencing a lack of interest or pleasure in activities (63.3%) and feelings of sadness, depression, or hopelessness for several days (71.3%). Most women had difficulty falling asleep or experienced excessive sleep, felt tired or had low energy levels, and had trouble concentrating on things for several days. Conversely, most of the participants reported no issues with appetite or overeating (58.5%), did not feel bad about themselves or consider themselves as failures (78.7%), exhibited normal movement and speech patterns (54.4%), and did not experience thoughts of self-harm (86.8%). Among the participants, the prevalence of depressive symptom 47.0%.

**Table 3:** Assessment of depressive symptoms among participants.

Item	Not at all (%)	Several days (%)	More than half the days (%)	Nearly everyday (%)
1. Little interest or pleasure in doing things	128 (30.7)	264 (63.3)	25 (6.0)	–
2. Feeling down, depressed, or hopeless	66 (15.8)	297 (71.3)	53 (12.7)	1 (0.2)
3. Trouble falling or staying asleep, or sleeping too much	192 (46.1)	198 (47.5)	26 (6.2)	1 (0.2)
4. Feeling tired or having little energy	163 (39.1)	178 (42.7)	74 (17.7)	2 (0.5)
5. Poor appetite or overeating	244 (58.5)	145 (34.8)	26 (6.2)	2 (0.5)
6. Feeling bad about yourself or that you are a failure or have let yourself or your family down	328 (78.7)	65 (15.6)	23 (5.5)	1 (0.2)
7. Trouble concentrating on things, such as reading the newspaper or watching television	131 (31.4)	259 (62.1)	27 (6.5)	–
8. Moving or speaking so slowly that other people could have noticed? Or the opposite—being so fidgety or restless that you have been moving around a lot more than usual	227 (54.4)	171 (41.1)	18 (4.3)	1 (0.2)
9. Thoughts that you would be better off dead or of hurting yourself in some way	362 (86.8)	38 (9.1)	17 (4.1)	–
Total PHQ-9 score (Mean±SD)	5.32 ± 2.93			

PHQ-9: Patient Health Questionnaire.

Overall sleep quality using the PSQI score and its components has been assessed and presented in Table 4. The global PSQI score was 5.77 ± 2.83, and about 36.9% of the participants reported poor sleep quality having a PSQI score of ≥ 5. Additionally, analysis of individual PSQI components revealed that majority of the participants reported fairly to very good subjective sleep quality (71.0%), had sleep latency up to 30 min (71.0%), had a sleep duration of more than 6 hours (87.3%), and had a habitual sleep efficiency of ≥85% (67.4%). Most of the participants did not use sleep medication during the past month (81.6%). A significant positive correlation (p < 0.05) was observed between the global PSQI score and each PSQI component, except for the associations of sleeping medication use and daytime dysfunction with habitual sleep efficiency (Table 5).

**Table 4:** Overall sleep quality and components among participants.

PSQI component	n (%)
Global PSQI score (Mean±SD)	5.77 ± 2.83
Subjective sleep quality (Mean±SD)	1.15 ± 0.80
*Very good*	83 (19.9)
*Fairly good*	213 (51.1)
*Fairly bad*	95 (22.8)
*Very bad*	26 (6.2)
Sleep latency (Mean±SD)	1.30 ± 0.88
* ≤ 15 min*	112 (26.9)
*16–30 min*	184 (44.1)
*31–60 min*	107 (25.6)
* > 60 min*	14 (3.4)
Sleep duration (Mean±SD)	0.98 ± 0.62
* > 7 h*	73 (17.5)
*6–7 h*	291 (69.8)
*5–6 h*	41 (9.8)
* < 5 h*	12 (2.9)
Habitual sleep efficiency (Mean±SD)	0.42 ± 0.69
* ≥ 85%*	281 (67.4)
*75–84%*	104 (24.9)
*65–74%*	24 (5.8)
* < 65%*	8 (1.9)
Sleep disturbance (Mean±SD)	1.12 ± 0.40
Use of sleeping medication (Mean±SD)	0.25 ± 0.58
*Not during the past month*	340 (81.6)
* < 1 a week*	56 (13.4)
*1–2 a week*	16 (3.8)
* ≥ 3 a week*	5 (1.2)
Daytime dysfunction (Mean±SD)	0.55 ± 0.67

PSQI: Pittsburgh Sleep Quality Index, SD: Standard deviation.

**Table 5:** Correlation between overall sleep quality and each component score.

PSQI component	1	2	3	4	5	6	7
1. Subjective sleep quality	1						
2. Sleep latency	0.545^**^	1					
3. Sleep duration	0.282^**^	0.222^**^	1				
4. Habitual sleep efficiency	0.203^**^	0.279^**^	0.360^**^	1			
5. Sleep disturbance	0.425^**^	0.316^**^	0.114^*^	0.159^**^	1		
6. Use of sleeping medication	0.273^**^	0.252^**^	0.166^**^	0.021	0.174^**^	1	
7. Daytime dysfunction	0.342^**^	0.304^**^	0.147^**^	-0.006	0.188^**^	0.368^**^	1
Global PSQI score	0.763^**^	0.751^**^	0.542^**^	0.493^**^	0.505^**^	0.514^**^	0.561^**^

PSQI: Pittsburgh Sleep Quality Index. ^*^Correlation is significant at the 0.05 level (2-tailed), ^**^Correlation is significant at the 0.01 level (2-tailed).

Table 6 shows the level of sleep quality based on anxiety and depressive symptoms among participants. It was observed that participants with anxiety symptoms had significantly poorer sleep quality compared to those without anxiety symptoms (6.07 vs. 5.39, p = 0.015). Similarly, participants with depressive symptoms had higher PSQI scores (6.23 ± 2.87) than the participants without symptoms (5.26 ± 2.69).

**Table 6:** Relationship of overall sleep quality scores with anxiety, and depressive symptoms among participants.

Variable		Mean (SD)	t	Sig.^*^
Anxiety symptoms	No	5.39 (2.81)	-2.439	0.015
	Yes	6.07 (2.81)		
Depressive symptoms	No	5.26 (2.69)	-3.528	<0.001
	Yes	6.23 (2.87)		

SD: Standard deviation. ^*^Independent sample t-test.

The association of sleep quality with anxiety and depressive symptoms among WRA using the linear regression models is depicted in Table 7. The multicollinearity between anxiety and depression scores was measured using the Variance Inflation Factor (VIF), and a value of <5 was noted, indicating the results were valid. Both anxiety and depressive symptoms remained significantly associated with sleep quality in the adjusted model. Results revealed that a unit increase in anxiety and depressive symptoms score was associated with a 0.243 (p < 0.001) and 0.285 (p < 0.001) point increase in PSQI score (decreased sleep quality), respectively.

**Table 7:** Regression analysis for sleep quality explained by anxiety, and depressive symptoms among participants.

Variable	Unadjusted Model			Adjusted Model^§^		
	*β*	95% CI	Sig.	*β*	95% CI	Sig.
Anxiety symptoms	0.242	0.208, 0.276	<0.001	0.243	0.209, 0.277	<0.001
Depressive symptoms	0.298	0.264, 0.332	<0.001	0.285	0.252, 0.318	<0.001

*β*: Standardized coefficient. CI: Confidence interval. ^**§**^The regression model was adjusted for age, residence, marital status, level of education, occupation, monthly household income, family size, BMI, MDD-W, and physical activity.

## Discussion

In the context of increasing mental health concerns among WRA, the present study aimed to assess the prevalence of anxiety and depressive symptoms, and poor sleep quality, as well as to examine the influence of anxiety and depression on sleep disturbances among reproductive-aged women in Noakhali, Bangladesh. The present study observed a relatively high prevalence of anxiety and depressive symptoms, and poor sleep quality among the participants. Furthermore, statistical analysis revealed a significant association between mental health conditions (anxiety and depression) and impaired sleep quality within the study population.

The present study found a prevalence of anxiety and depressive symptoms among the participants approximately 44.6% and 47%, respectively. These prevalences are substantially higher than those reported in prior national and international studies [3,22,38]. It is important to note that the relatively higher prevalence of anxiety and depressive symptoms observed in this study may partly reflect the lower cutoff (≥5) applied to the GAD-7 and PHQ-9 scales, which captures mild as well as moderate-to-severe cases. In contrast, several previous studies, used a cutoff of ≥7 or ≥10 to report moderate or severe symptoms. This methodological difference should be considered when comparing prevalence estimates across studies.

The finding of this study revealed that 36.9% of participants reported poor sleep quality, as assessed by the PSQI scale. This prevalence is relatively consistent with prior studies [22,39]. Besides, the current study observed a lower prevalence compared to several other studies conducted during heightened stress periods or in different socio-cultural contexts, for instance, in Bangladesh [19,35,40], Ethiopia [41], Canada [42], and Brazil [43]. Conversely, the findings of this study exceed those of Thalib et al. [44] who identified a 27.5% prevalence among Saudi Arabian women aged 18–60 years using the PSQI. However, differences in study timing, sample characteristics, and methodological variations - such as data collection mode (online vs face-to-face), measurement period (pre-, during-, or post-pandemic), and age distribution may account for the observed variations with the present study [45].

The relatively high prevalence of anxiety, depressive symptoms, and poor sleep quality observed in this study may also be influenced by the socio-cultural and environmental context of Noakhali. As a predominantly rural and coastal district, Noakhali frequently experiences economic hardship, livelihood instability, and seasonal disruptions due to flooding and cyclones, all of which can elevate psychological stress among women. Additionally, traditional gender roles, limited autonomy in household decision-making, and restricted access to mental health resources may further exacerbate anxiety and depressive symptoms. Cultural stigma surrounding mental health issues may also discourage help-seeking behaviors, allowing emotional distress to persist untreated. These contextual factors likely interact to increase vulnerability to both mental health problems and sleep disturbances among WRA in this setting [46]. A recent study from the United States by Balsara et al. [47], examining depressive symptoms through the lens of allostatic load and income inequality, also reinforces the importance of socio-structural determinants in shaping mental-health outcomes. Although conducted in a high-income setting, the study highlights a similar pathway to the one observed in the present low- and middle-income country context, where structural vulnerabilities and socioeconomic constraints contribute to psychological vulnerability.

This study revealed a significant association of sleep quality with both anxiety and depressive symptoms among WRA, suggesting that anxiety and depressive symptoms was associated with the occurrences of poor sleep quality. The adverse effect of anxiety and depression on sleep quality was also observed in the previous studies conducted in Bangladesh along with other countries instead of the variation in using scales to assess anxiety, depression, and sleep quality [18,20,22,48].

Empirical scientific literature has already established the multifactorial linkage of anxiety and depression with sleep quality (both short- and long-term sleep disturbances) including neurobiological, behavioral, and psychological mechanisms. These associations are particularly significant among WRA, a group uniquely vulnerable due to hormonal fluctuations, psychosocial stressors, and reproductive health concerns. In detail, excessive anxiety activates the hypothalamic-pituitary-adrenal axis which further enhances the cortisol levels and causes hyperarousal [49]. It becomes difficult to fall and stay asleep for a person suffering from hyperarousal [50]. Similarly, neurotransmitter imbalances, such as reduced serotonin and dopamine in depression, impair the regulation of sleep cycles, often resulting in insomnia, fragmented sleep, or hypersomnia [51]. Depression may also contributes to irregular sleep-wake cycles and persistent fatigue, all which may be compounded by hormonal changes specific to reproductive-age women [52].

The above disruptions further worsen mood disorders, creating a vicious cycle where poor mental health perpetuates poor sleep [48]. This cyclical interaction may be especially detrimental in WRA, who often navigate additional stressors related to fertility, pregnancy, or parenting [53]. Furthermore, a review by Medic et al. [54] reported that acute and persistent sleep disturbances may contribute to gastrointestinal disorders, including inflammatory bowel disease, irritable bowel syndrome, gastroesophageal reflux disease, impaired cognitive function, work performance, and even suicidal ideation, particularly among vulnerable groups such as women during reproductive years. Considering the significant associations observed, interventions targeting anxiety and depression hold promise for improving sleep quality in this demographic. Addressing the root psychological causes may therefore be a crucial step toward enhancing both mental and physical health outcomes among women of reproductive age.

## Public health implication

The findings of this study underscore the critical public health need to address mental health challenges - particularly anxiety and depressive symptoms - among WRA in Bangladesh. Given the significant association observed between poor sleep quality and psychological distress, integrating mental health services into primary healthcare and reproductive health programs is essential. Tailored interventions, including community-based awareness campaigns, mental health screening, and accessible counseling services, should be prioritized in rural and underserved regions like Noakhali. Additionally, improving health literacy and reducing stigma around mental health conditions can promote early identification and treatment, ultimately enhancing women’s quality of life, overall well-being, and active participation in economic, personal, and community life. Public health policies must also recognize sleep health as a critical component of women’s health, ensuring that interventions for mental well-being concurrently address sleep quality to foster holistic care.

## Limitations

This study has several limitations that should be considered when interpreting the findings. First, the cross-sectional design restricts the ability to establish causal relationships between anxiety, depression, and sleep quality. Second, the sample is not representative of all WRA in Bangladesh although a large number of subjects were included. Third, the reliance on self-reported measures such as the GAD-7, PHQ-9, and PSQI, although validated, may introduce recall bias and subjectivity in responses. Additionally, potential confounding variables such as comorbid physical illnesses, medication use, and stress levels were not accounted for, which could influence both mental health outcomes and sleep patterns. Furthermore, data regarding participants’ pregnancy or postpartum status were not collected. Given that both pregnancy and the postpartum period can substantially affect psychological well-being and sleep quality, the absence of this information limits our ability to control for these factors in the analysis. Moreover, anxiety and depressive symptoms may vary over time due to situational or seasonal influences such as workload changes, climatic variation, or social events. As data were collected during a single time period, the findings may not reflect potential temporal fluctuations in mental health or sleep quality across different seasons, thereby limiting generalizability over time. Finally, the absence of longitudinal follow-up restricts the understanding of temporal changes or potential long-term effects.

## Conclusion

This study highlights a considerable burden of mental health issues and poor sleep quality among WRA in Noakhali, Bangladesh. The results revealed that a high proportion of participants experienced anxiety and depressive symptoms as well as poor sleep quality. Importantly, both anxiety and depressive symptoms were significantly positively associated with poor sleep quality. Hence, the study findings emphasize the urgent need for integrated mental health and sleep health interventions, particularly in resource-limited rural settings. In this context, integrated interventions could include community health worker-led screening programs for anxiety, depression, and sleep problems; mobile mental health clinics providing counseling and referral services; and community-based awareness campaigns to reduce stigma and promote healthy sleep practices. Public health strategies aimed at early detection and management of anxiety and depression may contribute to improved sleep quality and overall well-being in this vulnerable population group. Future longitudinal studies and intervention-based research are warranted to explore causal relationships and develop effective, context-specific mental health support systems for women in Bangladesh.
